# Characterization of long noncoding RNA and messenger RNA signatures in melanoma tumorigenesis and metastasis

**DOI:** 10.1371/journal.pone.0172498

**Published:** 2017-02-22

**Authors:** Siqi Wang, Wenliang Fan, Bing Wan, Mengqi Tu, Feng Jin, Fang Liu, Haibo Xu, Ping Han

**Affiliations:** 1 Department of Radiology, Union Hospital of Tongji Medical College, Huazhong University of Science and Technology, Wuhan, People’s Republic of China; 2 Department of Radiology, The First Affiliated Hospital of Inner Mongolia Medical University, Hohhot, People’s Republic of China; 3 Department of Radiology, Zhongnan Hospital of Wuhan University, Wuhan, People’s Republic of China; University of Queensland Diamantina Institute, AUSTRALIA

## Abstract

The incidence of melanoma, the most aggressive and life-threatening form of skin cancer, has significantly risen over recent decades. Therefore, it is essential to identify the mechanisms that underlie melanoma tumorigenesis and metastasis and to explore novel and effective melanoma treatment strategies. Accumulating evidence s uggests that aberrantly expressed long noncoding RNAs (lncRNAs) have vital functions in multiple cancers. However, lncRNA functions in melanoma tumorigenesis and metastasis remain unclear. In this study, we investigated lncRNA and messenger RNA (mRNA) expression profiles in primary melanomas, metastatic melanomas and normal skin samples from the Gene Expression Omnibus database. We used GSE15605 as the training set (n = 74) and GSE7553 as the validation set (n = 58). In three comparisons (primary melanoma versus normal skin, metastatic melanoma versus normal skin, and metastatic melanoma versus primary melanoma), 178, 295 and 48 lncRNAs and 847, 1758, and 295 mRNAs were aberrantly expressed, respectively. We performed Gene Ontology and Kyoto Encyclopedia of Genes and Genomes pathway analyses to examine the differentially expressed mRNAs, and potential core lncRNAs were predicted by lncRNA-mRNA co-expression networks. Based on our results, 15 lncRNAs and 144 mRNAs were significantly associated with melanoma tumorigenesis and metastasis. A subsequent analysis suggested a critical role for a five-lncRNA signature during melanoma tumorigenesis and metastasis. Low expression of U47924.27 was significantly associated with decreased survival of patients with melanoma. To the best of our knowledge, this study is the first to explore the expression patterns of lncRNAs and mRNAs during melanoma tumorigenesis and metastasis by re-annotating microarray data from the Gene Expression Omnibus (GEO) microarray dataset. These findings reveal potential roles for lncRNAs during melanoma tumorigenesis and metastasis and provide a rich candidate reservoir for future studies.

## Introduction

The worldwide incidence of melanoma, the most aggressive form of skin cancer, has rapidly increased in recent decades. The number of new melanoma cases in the United States is expected to reach 76,380, with 10,130 deaths, by the end of 2016 [1]. Primary melanoma (PM) is curable by surgery. However, if it is not detected early and surgically removed, melanoma is highly likely to metastasize. Thus, identification of the mechanisms driving both tumorigenesis and metastasis and the development of novel and effective melanoma treatment strategies are urgently needed.

Long noncoding RNAs (lncRNAs), which exceed 200 nucleotides in length, are messenger RNA (mRNA)-like transcripts that do not encode proteins [2, 3]. Unlike smaller microRNAs, which play crucial roles in human carcinogenesis, our understanding of lncRNA biological functions is in its infancy. The first functional lncRNA, XIST, was discovered in the early 1990s; XIST inactivates gene expression from the X-chromosome by dosage equalization [4, 5]. Multiple reports have shown that lncRNAs regulate complex and diverse functions, including embryonic stem cell pluripotency [6], epigenetic gene regulation [7], the DNA damage response [8], and chromatin remodeling [9]. Furthermore, lncRNAs participate in wide-ranging cellular processes, including cell cycle, proliferation, apoptosis, and invasion [10].

With the emergence of next generation sequencing, large projects have identified multiple lncRNAs that are involved in carcinogenesis and development of cancer [11, 12], including glioblastoma [13], ovarian cancer [14], hepatocellular carcinoma [15], gastric cancer [16] and colorectal cancer (CRC) [17]; this knowledge suggests intriguing possibilities for diagnostic and therapeutic lncRNA applications. However, little is known about lncRNA functions in melanoma tumorigenesis and metastasis. Multiple studies have identified several functions for lncRNAs in melanoma. Upregulation of SPRY4-IT1 might play an important role in melanoma and be a useful early biomarker in humans [18, 19]. Tang et al. [20] has shown HOTAIR overexpression in lymph node metastases compared to PMs and demonstrated an active role in cell motility and invasion, which highlights HOTAIR as a potential target for malignant melanoma therapy. A long intergenic non-coding RNA, CASC15, correlates with melanoma progression and is involved in the regulation of phenotype-switching [21]. Another lncRNA, SLNCR1, promotes melanoma invasion by binding to the androgen receptor and brain-specific homeobox protein 3a [22]. Nevertheless, the low specificities and sensitivities of lncRNAs suggest that a single target is not likely to fully illustrate lncRNA mechanisms in melanoma. The potential roles of lncRNAs during melanoma tumorigenesis and metastasis have not yet been fully explored.

We began our study by analyzing previously published melanoma gene expression profiles from the Gene Expression Omnibus (GEO) database and conducted lncRNA profiling to identify significant lncRNAs. The identified lncRNA profiles were then verified using another independent validation set. A Gene Ontology (GO) analysis, Kyoto Encyclopedia of Genes and Genomes (KEGG) pathway analysis, and lncRNA-mRNA co-expression network analysis were then conducted. We performed survival analysis based on TCGA database. Our findings might uncover possible lncRNA and mRNA expression profiles associated with melanoma progression and metastasis and provide novel insights into the molecular pathogenesis of melanoma.

## Materials and methods

### GEO gene expression data

PM and metastatic melanoma (MM) gene expression data were obtained from publicly available GEO databases (GSE15605 and GSE7553). We followed a strategy utilizing the larger dataset (GSE15605) as the training set and another independent dataset (GSE7553) as the validation set [23]. All of the samples in these datasets were hybridized with the HG-U133 Plus 2.0 Array (Affymetrix, Santa Clara, CA, USA); this array includes 54,675 probe sets and is widely used in biological research.

### lncRNA annotation pipeline

To evaluate lncRNA expression in the melanoma gene expression data, we applied an lncRNA annotation pipeline based on the method constructed by Zhang et al. [24]. First, the Affymetrix HG-U133 Plus 2.0 probe set ID was mapped to the latest version of the NetAffx Annotation File (**S1 Table,** HG-U133_Plus_2 Annotations, CSV format, Release 34, 30 MB, 1/23/14). The annotations contained the probe set ID, gene title, gene symbol, Ensembl, Refseq transcript ID and other information. Second, for the probe sets from the Refseq database, the IDs that were labeled “NR” were retained (NR indicated non-coding RNA). For the probe sets from the Ensembl database, the IDs with “antisense”, “processed transcripts”, “sense-overlapping”, “non_sense_mediated_decay”, “sense_intronic”, “lincRNA”, “non-coding”, “misc-RNA” or “3prime-overlapping-ncrna” in the Ensembl annotations were retained. Of the probe sets from the Refseq and Ensembl databases, those that were labeled “NR” in the Refseq database and also annotated with the above Ensembl gene titles were retained. Third, the probe sets were filtered by removing pseudogenes, microRNAs, rRNAs and other small RNAs, including snRNAs, snoRNAs, and tRNAs.

### Microarray data processing and differential expression analysis

All of the raw microarray CEL files were background-adjusted, normalized, and log-transformed using the Robust Multichip Average in the Affy package of the R software [25]. Differentially expressed lncRNAs and mRNAs that were involved in three comparisons were identified by the limma package [26] in the R software. The Benjamini-Hochberg false discovery rate was used to correct the P values [27]. The threshold was an absolute log_2_ fold change (FC)>2 and p<0.05 for differentially expressed mRNAs and an absolute log_2_ FC>1 and p<0.05 for differentially expressed lncRNAs (Student’s t-test). The hierarchical clustering analysis was processed by Cluster3.0 & Treeview (Stanford University) [28].

### Co-expression network construction

The lncRNA-mRNA co-expression network was constructed based on the Pearson correlation coefficient (PCC) analysis between the lncRNA and mRNA expression levels. The PCC was calculated for each lncRNA-mRNA pair using MATLAB R2012a (MathWorks, Natick, MA, USA), and significant lncRNA-mRNA pairs with p<0.05 were selected to construct the co-expression network using the Cytoscape 3.4.0 program. The degree was defined as the number of directly linked neighbors. We further validated lncRNA-mRNA interactions in a database developed by Terai et al. [29] (http://rtools.cbrc.jp/cgi-bin/RNARNA/index.pl), which included all the predicted RNA-RNA interactions using 23,898 lncRNA and 81,814 mRNA sequences obtained from the Gencode project (http://www.gencodegenes.org/releases/19.html).

### GO enrichment and KEGG pathway analysis of lncRNA-co-expressed mRNAs

The GO and KEGG pathway analyses were conducted using DAVID (http://david.abcc.ncifcrf.gov/). The GO terms and the KEGG pathways with p<0.05 were selected to be the enriched functions of the differentially expressed mRNAs. GO analyses covered three domains: Biological Process, Cellular Component and Molecular Function.

### Kaplan–Meier analysis

The lncRNA reads per kilobase per million mapped reads (RPKM) of 221 skin samples from patients with cutaneous melanoma were downloaded from The Atlas of Noncoding RNAs in Cancer [30] (TANRIC, http://ibl.mdanderson.org/tanric/_design/basic/index.html), and corresponding clinical parameters and follow-up information for these patients were downloaded from The Cancer Genome Atlas (TCGA) [31]. Kaplan–Meier analyses were performed in R software to explore the association between the lncRNA and overall survival of patients with melanoma. According to the median level of each lncRNA expression, we divided patients with melanoma into low and high lncRNA expression groups; p< 0.05 was considered significant.

## Results

### GEO data set characteristics

**Fig 1:**
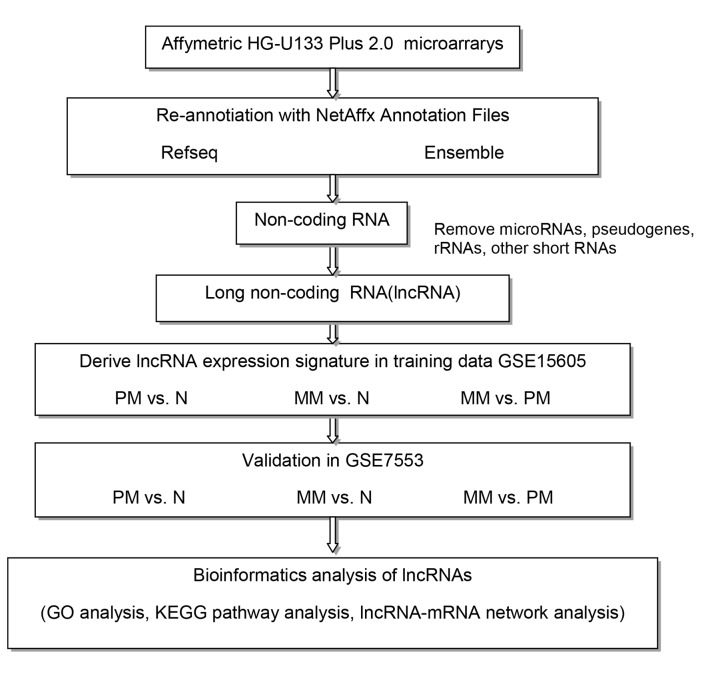
Study workflow. Abbreviations: PM, primary melanoma; MM, metastatic melanoma; N, normal skin.

The GSE15605 and GSE7553 series were obtained from GEO and used in this study. GSE15605 comprised 74 samples, including 46 PM, 12 MM, and 16 normal skin (N) samples. GSE7553 contained 87 samples, including 14 PM, 40 MM, 4 N samples, 15 basal cell carcinoma, 11 squamous cell carcinoma, 2 melanoma in situ, and 1 normal human epidermal melanocytes. For GSE7553, only 14 PM, 40 MM and 4 N samples were retrieved and analyzed. We used the larger data set (GSE15605) as the training set to detect gene expression signatures and GSE7553 as the validation set to confirm the results. Fig 1 depicts our experimental workflow.

### lncRNA expression profiles on Affymetrix HG-U133 Plus 2.0 arrays

We collected 3,805 probe sets (matched with 3,051 lncRNAs) by re-annotation of the Affymetrix HG-U133 Plus 2.0 arrays based on the NetAffx annotation and the Refseq and Ensembl databases (**S2 Table**). Of these, 1,470 probe sets (952 lncRNAs) were annotated as lncRNAs by both the Refseq and the Ensembl databases; 260 probe sets (215 lncRNAs) were annotated only by the Refseq database, and 2075 probe sets (1884 lncRNAs) were annotated only by the Ensembl database.

### Differentially expressed lncRNAs between PM, MM and N

**Fig 2:**
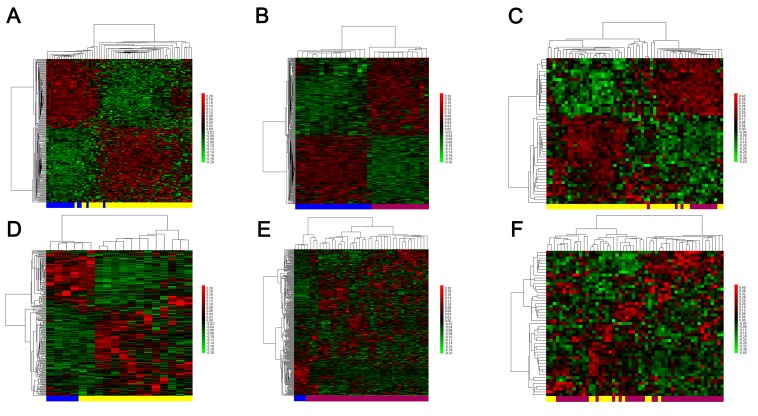
**Hierarchical clustering of differentially expressed lncRNA probe sets in PM vs. N (A, D), MM vs. N (B, E), and MM vs. PM (C, F).** The analyses were initially performed with the GSE15605 training set (A, B, C) and then validated in GSE7553 (D, E, F). Each row represents one lncRNA probe set, and each column represents one sample. High relative expression is indicated in red; low relative expression is indicated in green. The bar colors represent the sample types as indicated: blue, normal skin; yellow, primary melanoma; red, metastatic melanoma. Abbreviations: PM, primary melanoma; MM, metastatic melanoma; N, normal skin.

Using the absolute log_2_ FC>1 and p<0.05 threshold, differentially expressed lncRNAs were identified for the following three comparisons: PM vs. N, MM vs. N, and MM vs. PM. In PM vs. N, we identified 207 probe sets (178 lncRNAs) containing 108 upregulated probe sets (91 lncRNAs) and 99 downregulated probe sets (87 lncRNAs) (**S3 Table**). In MM vs. N, we identified 348 probe sets (295 lncRNAs) containing 185 upregulated probe sets (150 lncRNAs) and 163 downregulated probe sets (145 lncRNAs) (**S4 Table**). In MM vs. PM, we identified 51 probe sets (48 lncRNAs) containing 20 upregulated probe sets (17 lncRNAs) and 31 downregulated probe sets (31 lncRNAs) (**S5 Table**), as shown in Table 1. A hierarchical clustering analysis of all samples from the GSE15605 training set and the GSE7553 validation set were processed from these differentially expressed lncRNAs. The hierarchical clustering maps for the three comparisons revealed non-random partitioning of the samples into two major groups in GSE15605. Using a training-validation approach, we validated our results in the GSE7553 dataset. Similar distinctions between two sample types were observed (Fig 2).

**Table 1:** Number of differentially expressed lncRNAs and mRNAs in PM/N, MM/N, and MM/PM.

	lncRNA (p<0.05 | log_2_ FC|>1)			mRNA (p<0.05 | log_2_ FC|>2)		
	PM/N	MM/N	MM/PM	PM/N	MM/N	MM/PM
**Up**	108(91)	185(150)	20(17)	275(211)	677(517)	51(42)
**Down**	99(87)	163(145)	31(31)	803(636)	1638(1243)	296(253)
**Total**	207(178)	348(295)	51(48)	1078(847)	2315(1758)	347(295)

Abbreviations: PM, primary melanoma; MM, metastatic melanoma; N, normal skin. Probe set number (corresponding lncRNA number)

To examine the robustness and accuracy of differentially expressed lncRNAs, we searched for previously observed melanoma-associated lncRNAs in PubMed database. These lncRNAs included SAMMSON [32], HOTAIR [20], SLNCR1 [22], BANCR [33], SPRY4-IT1 [18], ANRIL [34], Llme23 [35], UCA1 [36], MALATA1 [36], GAS5 [37], H19 [38], CASC15 [21], PTENP1 [39], and MIR31HG [40]. Llme23 was not included in our lncRNA list. Most of the searched lncRNAs were consistent with our results, which indicated the accuracy and robustness of our study (S10 Table).

### Differentially expressed mRNA profiles between PM, MM and N

In PM vs. N, 1,078 probe sets (847 mRNAs) were identified as differentially expressed. Of these, 275 probe sets (211 mRNAs) were upregulated, and 803 probe sets (636 mRNAs) were downregulated (**S6 Table**). In MM vs. N, 2,315 probe sets (1,758 mRNAs) were identified as differentially expressed. Of these, 677 probe sets (517 mRNAs) were upregulated, and 1,638 probe sets (1,243 mRNAs) were downregulated (**S7 Table**). In MM vs. PM, 347 probe sets (295 mRNAs) were identified as differentially expressed. Of these, 51 probe sets (42 mRNAs) were upregulated, and 296 probe sets (253 mRNAs) were downregulated (**S8 Table**). The number of downregulated mRNAs was noticeably larger than the number of upregulated mRNAs (Table 1).

### lncRNA classification and distribution

**Fig 3:**
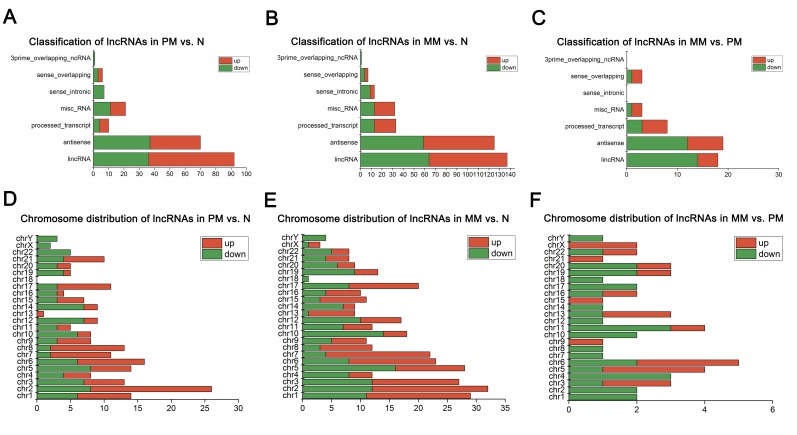
Chromosome distribution and classification of upregulated and downregulated lncRNAs in three comparisons. (A-C) Classification of lncRNAs in PM vs. N, MM vs. N, and MM vs. PM. (D-F) Chromosome distribution of lncRNAs in PM vs. N, MM vs. N, and MM vs. PM. The x-axis shows the number of lncRNAs. “Red” represents upregulated lncRNAs, and “Green” represents downregulated lncRNAs. Abbreviations: PM, primary melanoma; MM, metastatic melanoma; N, normal skin.

The differentially expressed lncRNAs were characterized as lincRNA, antisense, misc_RNA, processed_transcript, sense_intronic, sense_overlapping, and 3prime_overlapping_ncRNA. As shown in Fig 3A–3C, lincRNA and antisense were the two most common lncRNA biotypes in PM vs. N, MM vs. N, and MM vs. PM. Each chromosome contained different numbers of differentially expressed lncRNAs. In PM vs. N and MM vs. N, most lncRNAs were located on chromosome 2. Chromosome 18 had no lncRNAs in PM vs. N. In MM vs. PM, 7 lncRNAs (3 upregulated and 2 downregulated) were located on chromosome 6 (Fig 3D–3F).

### GO and KEGG pathway analyses

To further explore the potential functions of these differentially expressed mRNAs, we performed GO and KEGG pathway analyses. Upregulated and downregulated mRNAs were separately analyzed in the GO analysis and included the following three domains: Biological Process (GOBP), Cellular Component (GOCC) and Molecular Function (GOMF).

In PM vs. N, the top enriched GO terms among upregulated mRNAs included immune response (GOBP), extracellular region (GOCC), and calcium ion binding (GOMF), whereas oxidation-reduction process (GOBP), extracellular exosome (GOCC), and structural molecule activity (GOMF) were the top enriched GO terms among downregulated mRNAs.

In MM vs. N, the top enriched GO terms among upregulated mRNAs included immune response (GOBP), plasma membrane (GOCC), and transcription factor activity, sequence-specific DNA binding (GOMF), whereas oxidation-reduction process (GOBP), extracellular exosome (GOCC), and calcium ion binding (GOMF) were the top enriched GO terms among downregulated mRNAs.

In MM vs. PM, the top enriched GO terms among upregulated mRNAs included transcription, DNA-templated (GOBP), nucleus (GOCC), and DNA binding (GOMF), whereas epidermis development (GOBP), extracellular exosome (GOCC), and structural molecular activity (GOMF) were the top enriched GO terms among downregulated mRNAs.

**Fig 4:**
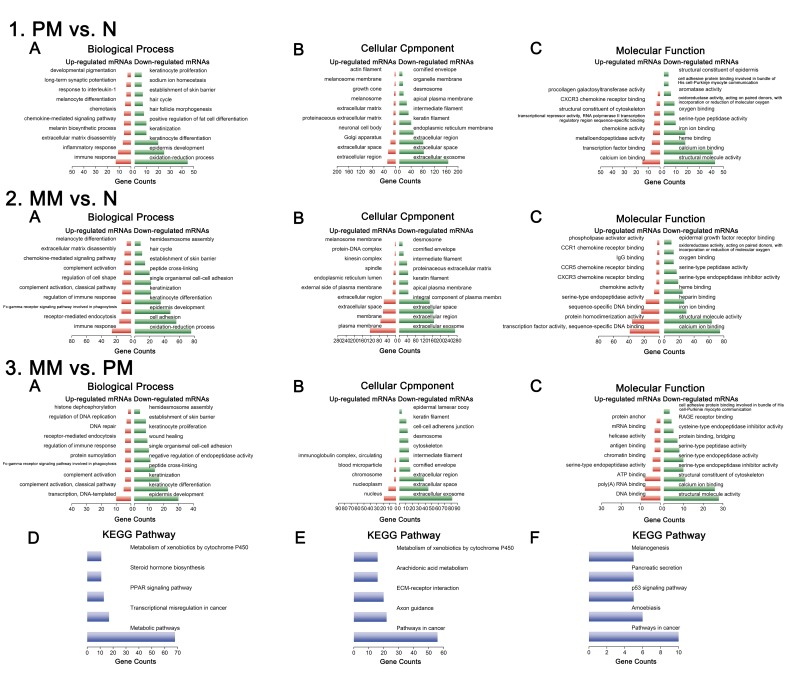
GO and KEGG pathway analyses of differentially expressed mRNAs. The GO analysis covers the following domains: (A) Biological Process; (B) Cellular Component; (C) Molecular Function. (D-F) KEGG pathway analysis: 1, PM vs. N; 2, MM vs. N; 3, MM vs. PM. p-value <0.05 is significant. The x-axis shows the number of mRNAs. Abbreviations: PM, primary melanoma; MM, metastatic melanoma; N, normal skin.

KEGG pathway analyses were also conducted for these three comparisons. The Metabolic pathways was the top enriched term in PM vs. N, and Pathways in cancer was the top enriched term in MM vs. N and MM vs. PM. The minimum ten GO terms and five pathway terms are shown in Fig 4 (with the exception of panel 4.3B and 4.3 C, which show a minimum of 5 and 9 terms that are statistically significant (p<0.05) for this set, respectively.)

### lncRNA-mRNA co-expression network analysis

**Fig 5:**
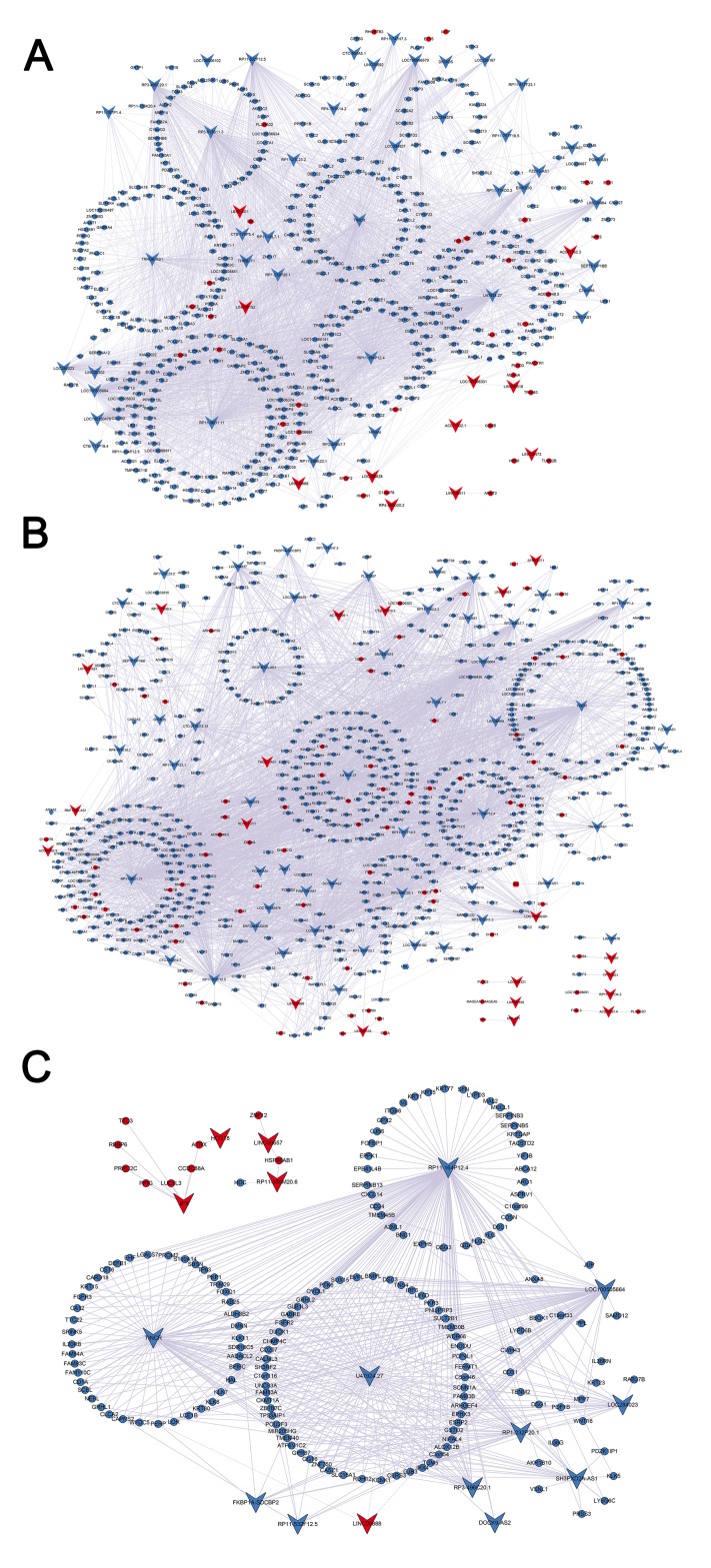
Co-expression network of differentially expressed lncRNAs and mRNAs. (A) The co-expression network was constructed with 54 differentially expressed lncRNAs and 472 associated mRNAs that were identified from PM vs. N. (B) The co-expression network was constructed with 73 differentially expressed lncRNAs and 666 associated mRNAs that were identified from MM vs. N. (C) The co-expression network was constructed with 16 differentially expressed lncRNAs and 169 associated mRNAs that were identified from MM vs. PM. The circular and v-shaped nodes are the mRNAs and lncRNAs, respectively. Upregulated genes are labeled in red; downregulated genes are labeled in blue. A solid line represents a positive correlation, and a dotted line represents a negative correlation. Abbreviations: PM, primary melanoma; MM, metastatic melanoma; N, normal skin.

We constructed an lncRNA-mRNA co-expression network to predict the potential functions of differentially expressed lncRNAs in PM and MM. Using a PCC analysis (absolute PCC>0.80, p<0.05), we identified 54 lncRNAs and 472 correlated mRNAs in PM vs. N to construct a clear network with 526 network nodes and 2402 connection edges (Fig 5A). The network indicated that multiple lncRNAs regulated numerous co-expressed mRNAs, including U47924.27, a downregulated lncRNA that was co-expressed with 235 mRNAs, and LINC00888, an upregulated lncRNA that was co-expressed with 15 mRNAs. In MM vs. N, we identified 73 lncRNAs and 666 correlated mRNAs by applying a PCC threshold of 0.90 and a significance threshold of 0.05. The network in MM vs. N contained 739 network nodes and 3145 connection edges (Fig 5B). Within this network, 2,866 pairs were positively correlated, and 278 pairs were negatively correlated. The network in MM vs. PM contained 185 network nodes (16 lncRNAs and 169 correlated mRNAs) and 459 connection edges (absolute PCC>0.80, p<0.05; Fig 5C).

### Venn diagram analysis

**Fig 6:**
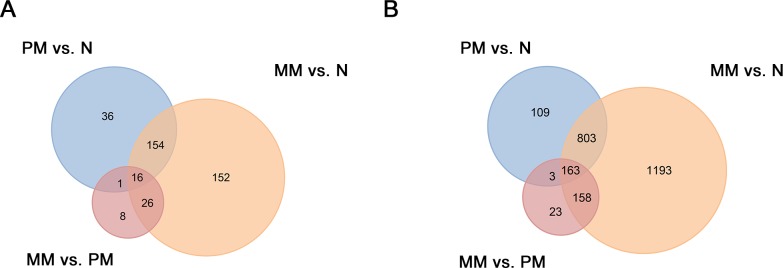
Venn diagram. (A) The number of lncRNA probe sets in the three groups. (B) The number of mRNA probe sets in the three groups. This figure depicts the number of common and unique probe sets in each comparison. The middle rectangle represents the number of lncRNA/mRNA probe sets that overlapped in all three comparisons. Abbreviations: PM, primary melanoma; MM, metastatic melanoma; N, normal skin.

To identify significantly expressed lncRNAs and mRNAs associated with melanoma tumorigenesis and metastasis, we constructed a Venn diagram analysis of the common and unique lncRNAs or mRNAs in the three comparisons (PM vs. N, MM vs. N, and MM vs. PM (Fig 6)). We identified 15 lncRNAs (16 probe sets) that overlapped in all three comparisons, including 12 downregulated lncRNAs and 3 upregulated lncRNAs (Table 2). As shown in Fig 6B, 144 mRNAs (163 probe sets) overlapped in all three comparison groups. Of these, 143 mRNAs were downregulated, including KRTDAP, KRT5, TACSTD2, and SERPINB5, but only SPP1 was upregulated (S9 Table).

**Table 2:** The 16 overlapped probe sets (15 lncRNAs) in PM/N, MM/N, and MM/PM.

Probe set ID	Gene symbol	log_2_ fold change		
		PM/N	MM/N	MM/PM
**Downregulated lncRNAs**				
228440_at	RP11-164P12.4	-2.349568	-4.883247	-2.480508
229385_s_at	TINCR	-3.030632	-5.259506	-2.308965
1557389_at	SH3PXD2A-AS1	-1.256226	-3.425267	-2.180685
242354_at	RP11-532F12.5	-2.862854	-4.970551	-2.076912
231089_at	LOC100505664	-2.300563	-4.111276	-1.772540
238498_at	RP3-406A7.7	-1.682248	-3.335783	-1.628080
240284_x_at	U47924.27	-2.660772	-4.268434	-1.504890
232300_at	ADIRF-AS1	-1.176617	-2.652849	-1.458243
240361_at	RP1-232P20.1	-2.164569	-3.529671	-1.387617
1558765_a_at	RP3-496C20.1	-2.077647	-3.343142	-1.373071
238096_at	LOC284023	-1.214494	-2.311894	-1.108923
232832_at	DKFZp434J0226	-1.391715	-2.503286	-1.066864
**Upregulated lncRNAs**				
228275_at	LINC00888	1.491120	2.861177	1.369500
1553608_a_at	LINC00189	1.055706	2.569488	1.494448
1566968_at	SPRY4-IT1	1.586614	3.081186	1.519854
1566967_at	SPRY4-IT1	1.646732	3.120109	1.522718

Abbreviations: PM, primary melanoma; MM, metastatic melanoma; N, normal skin.

**Fig 7:**
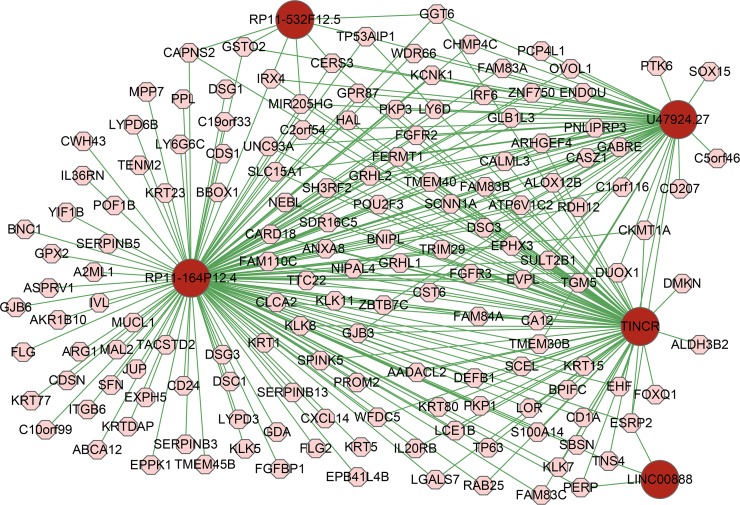
Co-expression network of five lncRNAs and co-expressed mRNAs. The co-expression network was constructed with 5 differentially expressed lncRNAs and 152 associated mRNAs that were identified from MM vs. PM. The red and pink nodes are the lncRNAs and mRNAs, respectively. Abbreviations: PM, primary melanoma; MM, metastatic melanoma.

Based on the log_2_ FC, p-value, and degree of associated mRNAs, we selected five lncRNAs, including RP11-164P12.4, TINCR, U47924.27, RP11-532F12.5 and LINC00888. We found mRNAs associated with these five lncRNAs accounted for a large proportion of the lncRNA-associated mRNAs (294/472, 467/666, and 152/169 in PM vs. N, MM vs. N, and MM vs. PM, respectively). We constructed a lncRNA-mRNA co-expression network with these five lncRNAs and associated 152 mRNAs in MM vs. PM, as shown in Fig 7. We further used a database developed by Terai et al. to analyze the target RNAs for these five lncRNAs. LINC00888 was not included in the database. The target RNAs for four lncRNAs are sorted by rank (using either MINENERGY or SUMENERGY). We showed the target mRNAs that overlapped in three comprisons (PM/N, MM/N, and MM/PM) of our study and the database developed by Terai et al. in S1 Fig and S11–S14 Tables. The results showed that most lncRNA coexpressed mRNAs in our study overlapped with target mRNAs in the database developed by Terai et al., which indicated the lncRNA-mRNA network in our study was worthwhile and accurate.

### Kaplan–Meier analysis

**Fig 8:**
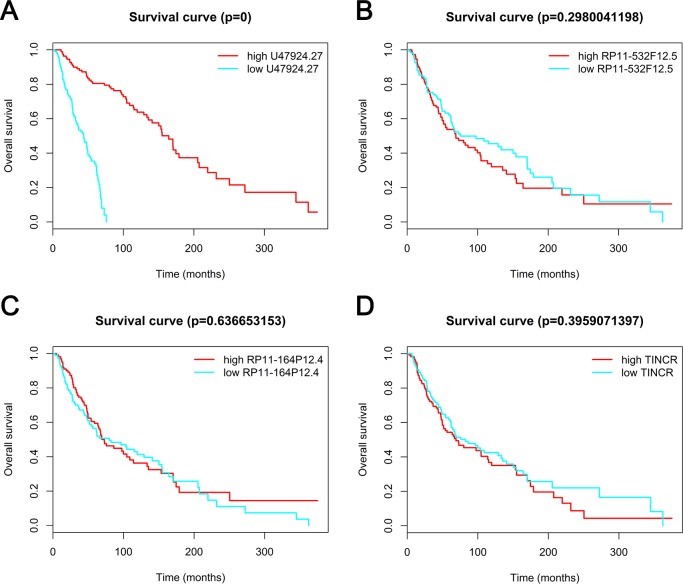
Survival analysis of patients with melanoma. Kaplan–Meier analyses were performed based on the median levels of U47924.27 (A), RP11-532F12.5 (B), RP11-164P12.4 (C), TINCR (D) expression. The expression of the lncRNAs was downloaded from TANRIC. The survival information was retrieved from TCGA.

We validated five lncRNAs, RP11-164P12.4, TINCR, U47924.27, RP11-532F12.5 and LINC00888, in the TCGA cohort containing 221 patients with melanoma. Four lncRNAs were annotated in the TCGA, and the findings showed that low expression of U47924.27 was associated with a shorter overall survival in patients with melanoma. However, a significant association was not detected for the other three lncRNAs (Fig 8).

## Discussion

Multiple recent studies have re-annotated microarray data to discover new lncRNA biomarkers and identify therapeutic lncRNA targets [24, 41, 42]. Zhang et al. investigated lncRNA expression profiles in gliomas by re-annotating the Affymetrix HG-U133 Plus 2.0 array [24]. This method permitted an lncRNA and mRNA expression analysis that was feasible, accurate, and inexpensive. Based on Zhang’s method, we explored lncRNA profiles in two existing melanoma patient cohorts from the GEO database as described [43, 44].

In our training set (GSE15605), we identified 178, 295, and 48 lncRNAs that were aberrantly expressed in PM vs. N, MM vs. N, and MM vs. PM, respectively. The validation of these results by another independent cohort (GSE7553) highlighted the usefulness of these lncRNA signatures.

Using Venn diagram analysis, we identified 15 aberrantly expressed lncRNAs that facilitated melanoma tumorigenesis and had vital functions in melanoma metastasis. Of these 15 lncRNAs, several have been reported in cancers, For example, LINC00189 participates in the tumorigenesis of squamous cell carcinoma of urinary bladder. However, only SPRY4-IT1 and TINCR have been reported to participate in melanoma.

Several studies have identified known lncRNAs that are abnormally expressed in melanoma [45–47], such as SAMMSON [32], HOTAIR [20], SLNCR1 [22], BANCR [33], SPRY4-IT1 [18], ANRIL [34], Llme23 [35], UCA1 [36], MALATA1 [36], GAS5 [37], H19 [38], CASC15 [21], PTENP1 [39], and MIR31HG [40]. Most of these lncRNAs were also identified in our study, showing the accuracy and robustness of our study. Since we focused on identifying lncRNAs that are abnormally expressed during melanoma tumorigenesis and metastasis, only SPRY4-IT1 was included in our final lncRNA signature. SPRY4-IT1 was upregulated in all three comparisons in our study, consistent with previous reports, which showing that SPRY4-IT1 was overexpressed in melanoma cell lines. Knockdown of SPRY4-IT1 causes defects in cell growth and decreases invasion and migration, implying that SPRY4-IT1 upregulation might play a central role in melanoma tumorigenesis and metastasis and could serve as a useful early biomarker in humans [18, 19].

Based on the log_2_ FC, p-value, and number of associated mRNAs, we established five candidate lncRNAs (TINCR, RP11-164P12.4, RP11-532F12.5, U47924.27, and LINC00888) that might play critical roles in melanoma tumorigenesis and metastasis.

Tissue differentiation-inducing non-protein coding RNA (TINCR) was previously reported to be upregulated in esophageal squamous cell carcinoma and might facilitate its development through an association with CLND7 and ANAX1 [48]. Zhang et al. [49] showed that downregulated TINCR promoted proliferation and metastasis in CRC by acting as a potential cancer suppressor gene. Sarkar et al. [46] suggested that a gain of ANCR and loss of TINCR might maintain keratinocyte progenitors in their undifferentiated states, resulting in melanoma tumorigenesis. This association should be investigated further. In our study, TINCR was significantly downregulated in PM vs. N (log_2_ FC = -3.031) and downregulated further in MM vs. N (log_2_ FC = -5.260). This suggests that TINCR might play a critical regulatory role in melanoma formation and metastasis.

No reports that address the roles of RP11-164P12.4, RP11-532F12.5, U47924.27, and LINC00888, but we can predict their functions by analyzing their associated mRNAs. RP11-532F12.5 was downregulated with a log_2_ FC of -4.971 in MM when compared with normal skin tissues. Interestingly, an analysis of the RP11-532F12.5 genomic locus showed that RHOV was its near coding gene (38 kb away). RP11-532F12.5 might regulate the expression of neighboring protein-coding genes and influence the development and progression of melanoma. RHOV, which is co-expressed with RP11-532F12.5 (PCC = 0.943 in MM vs. N), is an apoptosis-associated genes in the Rho GTPase family. As shown in the study by Mikhail et al. [50], RHOV is overexpressed in lung cancer cell lines and human NSCLC tumors, suggesting a possible role in NSCLC progression.

RP11-164P12.4 was the top lncRNA of the downregulated group (log_2_ FC = -2.481) and showed the largest degree (degree = 144) in MM vs. PM. CASZ1 is associated with the RP11-164P12.4 mRNA (PCC = 0.924 in MM vs. PM), which has been shown to suppress neuroblastoma cell growth in vitro and in vivo [51].

U47924.27, which is located on chromosome 12, is a lincRNA (long intergenic non-coding RNA) and had the second highest number of neighboring mRNAs in PM vs. N and MM vs. N (degree = 235 and 285, respectively). IRX4 is an mRNA neighboring U47924.27 in the lncRNA-mRNA network (PCC = 0.904 and 0.972 in PM vs. N and MM vs. N, respectively) and is a member of the Iroquois homeobox family, which is involved in carcinogenesis [52, 53]. Nguyen et al. showed that knockdown of IRX4 promotes prostate cancer cell growth, whereas overexpression of IRX4 suppresses prostate cancer cell growth [54].

LINC00888, which is located on chromosome 3, had the most co-expressed mRNAs among the upregulated lncRNAs in the three comparisons. In MM vs. N, LINC00888 (upregulated) co-expressed with 13 mRNAs, 11 downregulated and 2 upregulated. One example was BNC1 (basonuclin 1), a downregulated zinc-finger transcription factor with numerous known targets, and loss of BNC1 increases the metastatic potential of breast cancer [55]. LINC00888 is negatively correlated with BNC1, which implies a potentially active role in melanoma.

In our study, 144 differentially expressed mRNAs overlapped in all three comparison groups. Of these, the following mRNAs should be highlighted: MUCL1, DSC3, SERPINB5, CST6, and SPP1. MUCL1 (mucin-like 1), also known as SBEM, was first identified by Miksicek et al. [56]. Conley et al. [57] observed that HER2 regulated MUCL1 to promote breast cancer cell growth through the FAK/JNK signaling pathway. Valladares-Ayerbes et al. [58] showed that SBEM was detectable in bone marrow micrometastases of breast cancer patients by RT-PCR, which implied its potential utility as a bone marrow micrometastasis marker for breast cancer. These findings revealed a correlation between MUCL1 and cancer progression. DSC3 (Desmocollin 3), a member of the cadherin superfamily, has been associated with lymph node metastasis in oral squamous cell carcinoma [59]. Recently, Pan et al. [60] observed that the loss of DSC3 in prostate cancer predicted a poor prognosis; our study was consistent with these results. DSC3 was downregulated in PM compared to N (log_2_ FC = -3.068) and more downregulated in MM compared to N (log_2_ FC = -7.705). Maspin (SERPINB5) was identified as a cancer suppressor gene in 1994 by Zou et al. [61]. Numerous studies have demonstrated that maspin loss predicts possible metastasis and poor patient prognosis for prostate [62], cervical [63], and gastric cancers [64]. Our results showed that SERPINB5 expression was significantly decreased in PM vs. N, MM vs. N and MM vs. PM. Moreover, multiple studies have shown that CST6 encodes a secreted protein (Cystatin E/M) that suppresses metastasis. Jin et al. revealed that CST6 inhibited migration, invasion, and bone metastasis in breast cancer [65]. Another study demonstrated that CST6 overexpression decreased metastasis in prostate cancer [66]. Accordingly, our results showed CST6 downregulation in all three comparison groups. Secreted phosphoprotein 1 (SPP1, also called osteopontin) has functions in tumorigenesis, tumor progression, and metastasis in numerous cancers [67–69]. Liu et al. [68] showed that shRNA-mediated SPP1 suppressed the proliferation, migration, and invasion of human renal cancer ACHN cells by regulating MMP2 and MMP9. A recent study by Agrawal et al. showed that SPP1 was consistently upregulated in high-grade, invasive, and recurrent urothelial cancer cases [70]. In our study of 146 overlapping mRNAs over three comparisons, SPP1 was the only upregulated mRNA.

To explore the functions of the differentially expressed mRNAs in melanoma, we constructed GO and pathway analyses. Several GO terms from the upregulated mRNAs were related to immune and inflammatory responses, including immune response, inflammatory response, chemotaxis and regulation of immune response. However, several GO terms from the downregulated mRNAs were related to skin development, including epidermis development, keratinocyte, and keratinocyte differentiation.

According to the KEGG pathway analysis, mRNAs were targeted to pathway in cancer, metabolic pathways, melanogenesis, the p53 signaling pathway, and the PPAR signaling pathway, and others. Pathway in cancer was the top enriched term in MM vs. N and MM vs. PM, suggesting that our differentially expressed mRNAs are correlated with cancer. According to the study by Dowling et al. [71], metabolic pathways differed between melanoma *in situ* and invasive melanoma. Melanogenesis can be a pathogenic factor during melanoma progression. Thus, melanogenesis inhibition is a rational MM therapy approach [72]. Numerous reports have shown that the p53 signaling pathway controls cancer cell apoptosis and growth and is considered a key tumor suppressor in over half of all sporadic human cancers [73–75]. Peroxisome proliferator-activated receptors (PPARs) are nuclear hormone receptors with three isoforms: PPARa, PPARc, and PPARb/d. PPAR agonists, such as the thiazolidinediones, might be useful treatment modalities for malignant melanoma and melisma [76].

Furthermore, lncRNA data from the TCGA database were utilized to assess the correlation between lncRNA expression and the overall survival of patients with melanoma. Low U47924.27 expression was associated with a shorter overall survival in this study, indicating that U47924.27 down-regulation might be a potential marker of a poor prognosis.

Our study had several limitations. First, Affymetrix HG-U133 Plus 2.0 arrays included some, but not all, of the possible lncRNAs present. Second, distinct lncRNA expression patterns implied potential relationships to melanoma, but we do not have direct experimental evidence to support this hypothesis. We primarily focused our study on the value of bioinformatics-based analyses for discovering novel or important lncRNAs and mRNAs expressed during melanoma tumorigenesis and metastasis. Finally, the N sample size in our validation dataset (GSE7553) was not large and might have increased the bias in our analysis.

## Conclusions

We are the first to report the identification of lncRNA and mRNA expression patterns in melanoma tumorigenesis and metastasis by re-annotating microarray data from the GEO microarray dataset. We identified 15 lncRNAs and 143 mRNAs that are associated with melanoma tumorigenesis and metastasis. Based on the follow-up investigation revealed that a five-lncRNA signature might have a critical role in melanoma tumorigenesis and metastasis. Furthermore, based on the TCGA database, low U47924.27 expression was associated with a shorter overall survival. Our study might provide a candidate reservoir for future investigations of lncRNAs and mRNAs associated with melanoma tumorigenesis and metastasis; more extensive investigations will be performed in the future.

## Supporting information

S1 Fig The target mRNAs of four lncRNAs that overlapped between three comprisons (PM/N, MM/N, and MM/PM) of our study and the database developed by Terai et al. Red circle represents target mRNAs in the database developed by Terai et al. Yellow circle represents target mRNAs in PM vs. N in our study. Purple circle represents target mRNAs in MM vs. N in our study. Blue circle represents target mRNAs in MM vs. PM in our study. Abbreviations: PM, primary melanoma; MM, metastatic melanoma; N, normal skin.
(TIF)

S1 Table HU-U133_Plus_2.na34.annot. (ZIP)

S2 Table lncRNAs represented on the Affymetrix Human Genome U133 Plus 2.0 Array Based on annotations by Refseq and Ensembl. (XLSX)

S3 Table Upregulated and downregulated lncRNAs between primary melanomas and normal skin samples. (XLSX)

S4 Table Upregulated and downregulated lncRNAs between metastatic melanomas and normal skin samples. (XLSX)

S5 Table Upregulated and downregulated lncRNAs between metastatic and primary melanomas. (XLSX)

S6 Table Upregulated and downregulated mRNAs between primary melanomas and normal skin samples. (XLSX)

S7 Table Upregulated and downregulated mRNAs between metastatic melanomas and normal skin samples. (XLSX)

S8 Table Upregulated and downregulated mRNAs between metastatic and primary melanomas. (XLSX)

S9 Table The 144 overlapping mRNAs that were differentially expressed in the three comparisons. (XLSX)

S10 Table The lncRNA list in this study according to the reported melanoma-associated lncRNAs. (XLSX)

S11 Table Partial list of mRNAs interacting with ENST00000508664 (RP11-164P12.4), sorted by MinEnergy and SumEnergy. (XLSX)

S12 Table Partial list of mRNAs interacting with ENST00000448587 (TINCR), sorted by MinEnergy and SumEnergy. (XLSX)

S13 Table Partial list of mRNAs interacting with ENST00000537269 (U47924.27), sorted by MinEnergy and SumEnergy. (XLSX)

S14 Table Partial list of mRNAs interacting with ENST00000565315 (RP11-532F12.5), sorted by MinEnergy and SumEnergy. (XLSX)
