# Correction: Characterization of long noncoding RNA and messenger RNA signatures in melanoma tumorigenesis and metastasis

**DOI:** 10.1371/journal.pone.0181129

**Published:** 2017-07-06

**Authors:** Siqi Wang, Wenliang Fan, Bing Wan, Mengqi Tu, Feng Jin, Fang Liu, Haibo Xu, Ping Han

There is an error in affiliation 1 for authors Siqi Wang, Wenliang Fan, Bing Wan, Mengqi Tu, Feng Jin, Fang Liu, and Ping Han. Affiliation 1 should be: Department of Radiology, Union Hospital, Tongji Medical College, Huazhong University of Science and Technology, Wuhan 430022, China.

There is an error in affiliation 3 for author Feng Jin. Affiliation 3 should be: Department of Radiology, The First Affiliated Hospital of Inner Mongolia Medical University, Hohhot 010050, China.

There is an error in affiliation 3 for author Haibo Xu. Affiliation 2 should be: Department of Radiology, Zhongnan Hospital of Wuhan University, Wuhan 430071, China
