# Supplementary figures and images for: Characterization of long noncoding RNA and messenger RNA signatures in melanoma tumorigenesis and metastasis
Source: PLoS One. 2017 Feb 22;12(2):e0172498. doi: 10.1371/journal.pone.0172498 (PMC5321451; doi:10.1371/journal.pone.0172498)

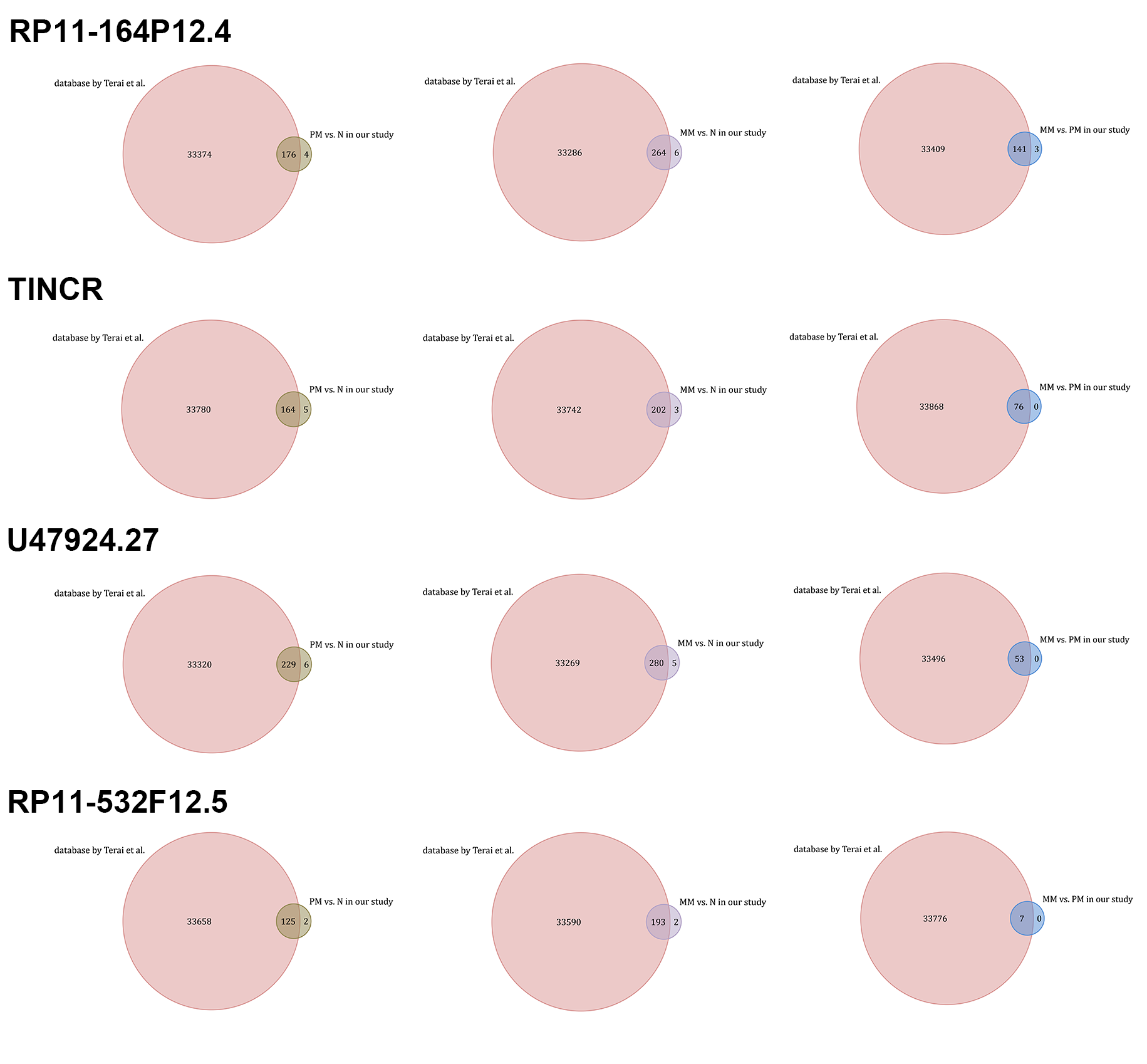

Supplement: S1 Fig — Red circle represents target mRNAs in the database developed by Terai et al. Yellow circle represents target mRNAs in PM vs. N in our study. Purple circle represents target mRNAs in MM vs. N in our study. Blue circle represents target mRNAs in MM vs. PM in our study. Abbreviations: PM, primary melanoma; MM, metastatic melanoma; N, normal skin. (TIF) [file pone.0172498.s001.tif]
